# Carlos Chagas Discoveries as a Drop Back to Scientific Construction of Chronic Chagas Heart Disease

**DOI:** 10.5935/abc.20160079

**Published:** 2016-07

**Authors:** Reinaldo B. Bestetti, Carolina Baraldi A. Restini, Lucélio B. Couto

**Affiliations:** Universidade de Ribeirão Preto, Ribeirão Preto, São Paulo, SP - Brazil

**Keywords:** Chagas Disease / history, Chagas Cardiomyopathy, Arrhythmias, Cardiac, Heart Failure, Carlos Chagas

## Abstract

The scientific construction of chronic Chagas heart disease (CCHD) started in 1910 when Carlos Chagas highlighted the presence of cardiac arrhythmia during physical examination of patients with chronic Chagas disease, and described a case of heart failure associated with myocardial inflammation and nests of parasites at autopsy. He described sudden cardiac death associated with arrhythmias in 1911, and its association with complete AV block detected by Jacquet's polygraph as Chagas reported in 1912. Chagas showed the presence of myocardial fibrosis underlying the clinical picture of CCHD in 1916, he presented a full characterization of the clinical aspects of CCHD in 1922. In 1928, Chagas detected fibrosis of the conductive system, and pointed out the presence of marked cardiomegaly at the chest X-Ray associated with minimal symptomatology. The use of serological reaction to diagnose CCHD was put into clinical practice in 1936, after Chagas' death, which along with the 12-lead ECG, revealed the epidemiological importance of CCHD in 1945. In 1953, the long period between initial infection and appearance of CCHD was established, whereas the annual incidence of CCHD from patients with the indeterminate form of the disease was established in 1956. The use of heart catheterization in 1965, exercise stress testing in 1973, Holter monitoring in 1975, Electrophysiologic testing in 1973, echocardiography in 1975, endomyocardial biopsy in 1981, and Magnetic Resonance Imaging in 1995, added to the fundamental clinical aspects of CCHD as described by Carlos Chagas.

## Introduction

Chagas disease affects about 6 million people in 21 countries of Latin American, where about 70 million people are at risk of acquiring the disease.^1^ The disease has been spread throughout the world because of immigration, with a global annual cost of about US 1 billion.^2^ Chagas disease is caused by the protozoan *Trypanosoma cruzi,* and is transmitted to humans through the feces of a hemipteran insect. Many years after infection, about 20% of patients develop chronic Chagas heart disease.^1^ This manuscript presents the history of the scientific construction of this heart disease from its discovery until modern days, emphasizing the main facts that contributed to the expansion of its knowledge over time. We do not allude to its pathophysiology, which is still debatable,^3^ or treatment aspects because they are not yet fully understood. Only studies published in full were added to the reference list.

## Chagas' period of study of the disease (1909-1934)

Carlos Chagas discovered the disease that bears his name in 1909. Details of this fascinating scientific achievement is described elsewhere.^4^ As early as 1910, Chagas emphasized the importance of the abnormalities in the cardiac rhythm found in some patients with chronic disease. Having into account data obtained in an autopsy case along with clinical findings, he suggested the existence of heart involvement in patients with chronic Chagas disease.^5^

**Figure 1:**
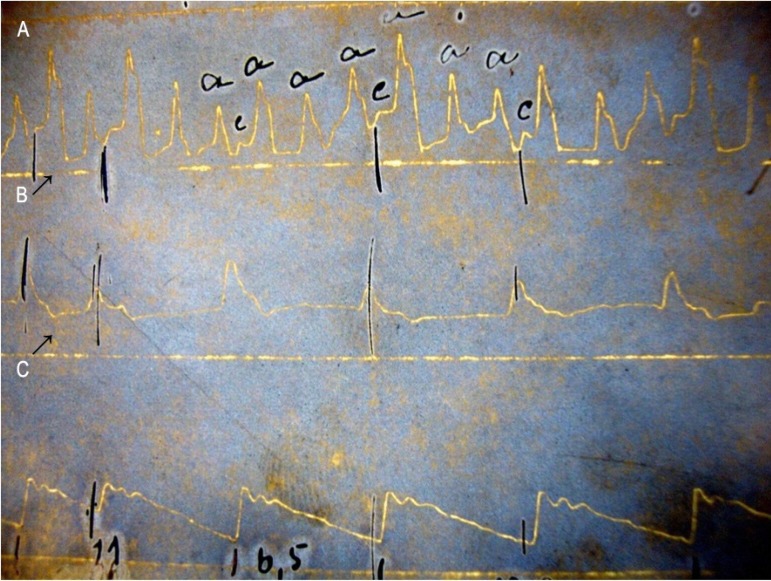
Original simultaneous jugular venous tracing (A), brachial artery tracing (B), and apexcardiogram (C) obtained with a Jacquet’s polygraph between 1910 and 1914 by Chagas team. Note a transitory second-degree AV block (arrows). The resting ECG was unavailable at that time in Brazil. a: A wave of the jugular pulse; c: C wave of the jugular pulse. Courtesy of Fundo Instituto Oswaldo Cruz, Seção Hospital Evandro Chagas, Acervo Casa de Oswaldo Cruz.

 Chagas emphasized the notorious presence of premature ventricular contractions (PVC) during physical examination, and stressed the presence of AV block, detected by Jacquet's polygraph (Figure 1) since electrocardiogram was not available at that time in Brazil, as a cause of abnormalities in cardiac rhythm. He presented the case of a patient with heart failure who was found to have nests of parasites accompanied by interstitial mononuclear cell infiltration in the myocardium at autopsy.^6,7^

In 1911, Chagas stated that such frequent episodes of arrhythmia on physical examination in individuals under 50 years were not observed outside endemic regions. In addition, he described the case of a patient with frequent PVC on physical examination who died suddenly; at autopsy, nests of parasites surrounded by severe mononuclear cell infiltration were found. Therefore, he suggested that PVC on physical examination could herald sudden death in the cardiac form of the chronic disease.^8^

In 1912, Chagas highlighted that disturbances in the cardiac conductibility could also be associated with sudden death. Furthermore, Chagas drew attention to a new fact: heart failure was the predominant clinical manifestation of the cardiac form, despite the presence of abnormalities in cardiac rhythm on physical examination. In addition, he pointed out the existence of profound bradycardia in some patients to the point of naming it "slow pulse disease".^9^ It is outstanding that such a clinical picture - arrhythmias and conduction disturbances in young people - was completely different from the heart diseases known at that time.^10^

In 1916, Chagas provided new findings on the histological aspects of patients with chronic Chagas heart disease. In addition to having mononuclear cell infiltration, he stated that all patients with this condition had important myocardial interstitial sclerosis (i.e fibrosis). Furthermore, Chagas ascribed the clinical picture of chronic Chagas heart disease- heart failure and arrhythmias- to these underlying myocardial lesions.^11^

In 1922, Chagas described the clinical characteristics of 62 patients with chronic Chagas heart disease studied from 1910 to 1921. Fifty patients (80%) had concomitant goiter, which might account, at least in part, for the fact that Chagas ascribed the same etiology to both conditions. A recent historical reappraisal suggests a relationship between both conditions.^12^ Palpitations and dyspnea were the most frequent complaints, and dysrhythmias, cardiac enlargement, and splitting of the second heart sound were frequently detected upon physical examination.

The cause of arrhythmias detected on physical examination by Jacquet's polygraph was PVC in 32 patients (51%), and atrioventricular AV block (second or third degree) in 20 (32%). In a time where serological reaction was not routinely available, such abnormalities in physical examination led Chagas to suggest their presence as a useful tool for the diagnosis of chronic Chagas heart disease. Heart failure was detected in 26 patients (42%). There were 16 deaths (16%); 50% of which were sudden. Of the sudden death cases, four were associated with advanced AV block. Thus, by 1922, Chagas had fully characterized the clinical and the morphological aspects of chronic Chagas heart disease.^13^

In 1928, with the availability of chest X-ray, Chagas could show a marked enlargement of the cardiac silhouette in a patient with only mild pedal edema, reinforcing the hallmark characteristic of heart failure in areas where the disease was endemic. Furthermore, Chagas provided histological pictures of patients with this condition for the first time, clearly showing the importance of inflammation and fibrosis in the myocardial and conductive tissue in the pathogenesis of chronic Chagas heart disease.^14^

In 1930, Evandro Chagas (Carlos Chagas' son) suggested the presence of right bundle branch block, which would be frequently diagnosed in patients with chronic Chagas heart disease in the following years, and that this intraventricular conduction disturbance could be associated with unfavorable prognosis in patients with the cardiac form of American Trypanosomiasis.^15^ Also in 1930, by using a serological reaction to confirm the diagnosis, Villela^16^ reported several patients with chronic Chagas heart disease in Belo Horizonte, Brazil, which was far from Lassance, the place where Chagas described the disease, thus suggesting that the disease could have a great epidemiological impact.

In 1931, Evandro Chagas documented the relentless progress of chronic Chagas heart disease with the use of the 3-lead ECG and chest X-ray, as Chagas had repeatedly stated based on clinical grounds. In 1934, taking into account the clinical course of the disease together with the histological abnormalities mentioned earlier, Chagas raised the possibility of an autoimmune mechanism in the pathogenesis of chronic Chagas heart disease.^17^

Thus, by 1934, Chagas had described the clinical picture with its peculiarities, including the presence of cardiac rhythm abnormalities to raise diagnostic suspicion, electrocardiographic abnormalities using Jacquet's polygraph, radiological features, detailed anatomopathological aspects, and possible pathogenetic mechanism of chronic Chagas heart disease. Apart from that, he had also described the etiology, vector, and reservoir of Chagas disease. The relentless progressive characteristic of the disease and its ominous prognosis were also apparent. Undoubtedly, it was the best that a scientist could do at that time.

## Forgetting a potential tragedy

In view of Carlos Chagas' work depreciation in Brazil and in Argentina, mainly at the School of Medicine of Rio de Janeiro, Oswaldo Cruz Institute, National Academy of Medicine,^4^ and Medical and Surgery Society of Rio de Janeiro,^18^ Chagas disease fell into oblivion of the medical profession following Chagas' death in 1934. It was no longer taught in medical schools.^19^ As a result, it was rarely diagnosed.^20^

The chronic phase of the disease started to be routinely diagnosed through serological reactions.^21^ By 1935, Chagas disease had already been recognized in at least four states of Brazil. In addition, the disease had also been detected in seven countries in Latin America, specially in Argentina.

## The disease regains credit in Brazil (1940-1945)

An important point in the construction of the clinical knowledge of chronic Chagas heart disease was the widespread use of the fixation complement test to make the serological diagnosis of the chronic disease. It was first reported by Guerreiro and Machado in 1913, but was considered impractical for widespread use for technical reasons.^22^ In 1936, Kelser^23^ modified the original fixation-complement reaction using antigens from artificial cultures of *T.cruzi*, which made it more stable. This made such reaction be used routinely in the diagnosis of chronic Chagas disease.^23^ Therefore, based on the clinical picture made up by Carlos Chagas years before, particularly in the presence of severe arrhythmias in young people originated from endemic areas, physicians started suspecting of chronic Chagas heart disease in several parts of Brazil, using either the xenodiagnosis or the serological reaction to confirm the diagnosis of this condition.

## The Bambui period (1945-1956)

The Bambui center (Minas Gerais state) greatly contributed to the determination of prevalence, incidence, and clinical course of Chagas disease in a large, unselected population of patients with this condition, by performing a systematic electrocardiographic survey in such patients, thus showing the enormous epidemiological importance of chronic Chagas heart disease.^24^

In 1945, Dias et al.^25^ reported the clinical findings of 90 new cases of chronic Chagas heart disease in Bambui. They emphasized the association of permanent splitting of the second heart sound in the pulmonary auscultation focus on physical examination - a sign already described by Chagas - with the presence of right bundle branch block in the resting ECG; lack of pulmonary vessel congestion in the presence of marked cardiomegaly in the chest X-Ray; high prevalence of 1^st^ degree AV block, highlighting its appearance early in the course of the disease and the importance of right bundle branch block observed in the 12-lead ECG for the diagnosis of this condition. Furthermore, they observed a marked left axis deviation of the QRS complex, later on diagnosed as left anterior hemiblock, associated with right bundle branch block. Dias et al.^25^ also drew attention to the variability of T wave inversion, disappearance of R wave in V4-V6 leads in association with left ventricular apical aneurysm, and the variability of QRS complexes and T waves from one tracing to another in cases of complete AV block.

The intermittence of complete AV block was described by Magalhães and Freire^26^ in 1945. A case of chronic heart failure with complete AV block complicated by pulmonary infarction was also reported for the first time in this year.^27^ In 1947, Pellegrino^28^ reproduced the clinical picture of chronic heart failure associated with the presence of right bundle branch block in the resting ECG of dogs chronically infected with T.cruzi. This fact lent support to the clinical picture described by Chagas and to those who were being reported by other authors at that time.^28^ In the same year, Rodovalho et al.^29^ emphasized the concomitance of left and right-sided heart failure in patients with chronic Chagas heart disease.

In 1948, Chiaverini^30^ reported his experience with 429 inpatients in São Paulo state with chronic heart disease, showing that chronic Chagas heart disease was the most frequent cause of heart disease in that hospital cohort, and that right bundle branch block was found more frequently in patients with this condition.^30^ Also in 1948, Barros^31^ observed that patients with chronic Chagas heart disease in the New York Heart Association Class (NYHA) II remained so for one to three years on average, and then progressed to NYHA Class IV, dying in 4 to 8 months on average. That was the first detailed clinical course of patients with chronic heart failure secondary to chronic Chagas heart disease. Barros also drew attention to the importance of hepatomegaly in the diagnosis of right-sided heart failure, which could precede the other physical signs observed in this condition.^31^

In 1953, Laranja^32^ pointed out that patients with chronic Chagas disease could remain in the indeterminate form of the disease for a long period, usually 10-15 years, thus providing evidence for the first time that a long time would elapse between the initial infection and the appearance of chronic Chagas heart disease.^32^ The coronation of the work at Bambui occurred in 1956. Laranja et al.^33^ presented clinical, epidemiologic, and pathologic data from a cohort of 1340 cases of chronic Chagas disease in which the 12-lead ECG was performed. They showed that 51% of patients presented abnormalities consistent with chronic Chagas heart disease in the resting ECG, thus establishing the prevalence of this condition in a large ambulatory population. Moreover, they showed that about half of chronic patients had the indeterminate form of the disease. Furthermore, Laranja et al.^33^ showed that out of the 40 patients with the indeterminate form of chronic Chagas disease with known end of the initial infection, 12 patients (30%) developed ECG alterations at 10-year follow-up. Therefore, they set the annual incidence of chronic Chagas heart disease at 3%.^33^

## The sixties

In 1964, based on experimental work, Rosenbaum^34^ stated that the peculiar electrocardiographic abnormalities observed in patients with chronic Chagas heart disease (right bundle branch block with left axis deviation) were consequence of widespread underlying chronic myocardial lesions. Rosenbaum^34^ named the left axis deviation as left anterior hemiblock. In 1965, his experience was reported in Brazil in the follow up of 86 patients with chronic Chagas heart disease. Twenty-seven patients (31%) died over a period of 10 years. Brazil observed 18 episodes of sudden death, thus yielding a yearly incidence of sudden death of about 3%. As observed by Chagas years before, complete AV block was the most important cause of sudden death. Thus, Brazil's findings contributed to the knowledge of the clinical course of sudden death due to chronic Chagas heart disease.^35^

### Heart catheterization

A patient with classic clinical, radiological, and electrocardiographic characteristics of this condition, as already reported in detail by the Bambui team, underwent heart catheterization in 1965.^36^ Mean atrial right pressure was 6 mmHg, right pulmonary systolic pressure 43 mmHg, pulmonary capillary wedge pressure 17 mmHg. This confirmed that chronic Chagas heart disease resembled the so called primary myocardial disease. Puigbó et al.^37^ studied 12 patients with this condition, but at the early stage of the disease (no heart failure, light or no cardiomegaly on the chest X-ray, but with abnormal resting ECG). Except for slight elevation of left ventricular end-diastolic pressure in 3 patients (25%), hemodynamic data were normal. Nonetheless, at left ventricular angiography, slight left ventricular dilatation was observed in 4 patients (33%), and left ventricular apical abnormalities in 10 (83%).^37^ This work was very important because it confirmed that a severe disease could occur in patients considered to be in the early stage of chronic Chagas heart disease, as Chagas had highlighted decades before.

## The seventies

### Refining the noninvasive diagnosis of chronic Chagas heart disease

#### Echocardiography

With the use of M-mode echocardiogram, it was possible to visualize abnormal heart anatomy and physiology noninvasively. Hernandez-Pieretti et al.^38^ reported findings obtained from five patients with chronic Chagas heart disease, namely: mitral movement compatible with low blood flow, thinning of the interventricular septum, paradoxical interventricular septal movement, hypokinesia/dyskinesia of the left ventricular posterior wall, dilatation of the left atrium, of the right ventricle, and of the left ventricle.^38^

One year later, asymptomatic patients showed segmental wall motion abnormalities either in the ventricular septum or in the left ventricular posterior wall, patients with moderate heart failure had abnormalities in interventricular septum contraction (hypokinesia, akinesia or paradoxical septal contraction), left ventricular hypokinesia, and right and/or left ventricular dilatation. In addition to the type of abnormalities mentioned earlier, patients with severe heart failure also had left ventricular systolic dysfunction and increased end-diastolic left ventricular pressure. Therefore, the way to measure the left ventricular systolic function noninvasively was created, which would be paramount in the following years.^39^

#### Exercise stress testing

Chagas had fiercely emphasized that many patients with chronic Chagas heart disease experienced sudden cardiac death while working as rural farmers. However, in 1973, Macedo et al.^40^ failed to reveal abnormalities in the exercise stress testing in patients in the indeterminate form of chronic Chagas disease, thus suggesting that Chagas disease patients with no heart involvement could perform rural work with no additional risk.^40^ Exercise stress testing, however, showed that work capacity was lower than expected in patients with chronic Chagas heart disease. In those with PVC in resting ECG, exercise stress testing aggravated the severity of such arrhythmias in many patients. Therefore, it became clear that exercise could either elicit or aggravate previous PVC with minimal physical limitation in patients with chronic Chagas disease.^41^

#### Holter monitoring

The electrocardiographic monitoring constituted a valuable tool for studying patients with chronic Chagas heart disease, not only in view of the high frequency of sudden cardiac death ascribed to ventricular dysrhythmias and AV conduction disturbances observed in this population, but also because of the intermittence of such abnormalities in patients with this condition. In fact, Hernandez-Pieretti et al.^42^ observed VPC in 5 of 17 patients (29%), sustained ventricular tachycardia in 3 (18%), 2^nd^. degree AV block in 2 (12%), complete AV block, and non-sustained ventricular tachycardia in 1 patient (6%).

#### Electrophysiological study

The introduction of electrophysiological study (EPS) into clinical practice in the late sixties was of enormous practical value for patients with chronic Chagas heart disease in view of the high frequency of bradyarrhythmias and fascicular blocks found in this disease. Benchimol et al. used EPS to diagnose a case of permanent atrial standstill and the presence of sinus node dysfunction in patients with chronic Chagas heart disease.^43,44^ Another important clinical component of sinus node dysfunction was the syndrome bradycardia-tachycardia, which was diagnosed with the EPS for the first time by Pimenta et al.^45^ in a patient with chronic Chagas heart disease. In a subsequent paper, Pimenta et al.^46^ showed that 28% of patients with chronic Chagas heart disease undergoing EPS were found to have abnormal AV nodal function. This highlighted the importance of the potential participation of both sinus node dysfunction and AV nodal dysfunction in the genesis of arrhythmias in patients with this condition.^46^

## The eighties

Acquatella et al.^47^ first described 64 patients with chronic Chagas heart disease at 2-D echocardiography. They observed a left ventricular apical aneurysm in 46% of patients, including those with the indeterminate form. Therefore, Acquatella et al. opened a window to the *in vivo* study of the anatomopathological aspects of this disease.^47^

The appearance of endomyocardial biopsy in the clinical scenario could potentially lead physicians to make morphological observations *in vivo,* and to pathological changes similar to those already described by Chagas in autopsy material. Mady et al.^48^ observed edema, slight inflammatory infiltrate, and myocardial fibrosis in patients with the indeterminate form of chronic Chagas disease.^48^ Subsequently, the same group expanded their findings by studying 42 patients with chronic Chagas disease, 16 with normal ECG and no cardiomegaly at chest X-Ray, 15 with abnormal ECG but normal chest X-Ray, and 11 with abnormalities in the ECG and in the chest X-Ray. They observed that the incidence of myocardial inflammation, myocardial hypertrophy, and myocardial fibrosis was higher in patients with abnormalities in the electrocardiogram as well as in the chest X-Ray. This study, therefore, confirmed the progressive nature of the disease *in vivo*.^49^

With the use of radionuclide study, it was also possible to establish the left ventricular ejection fraction noninvasively and to show wall motion abnormalities of the left ventricle, particularly in the infero-apical region.^50^ In 1985, Espinosa et al.^51^ provided new insight into the clinical course of patients with chronic Chagas disease, adding new evidence regarding disease progression. Patients with normal resting ECG and SWMA had a survival similar to that found in individuals in control groups; by contrast, those patients with abnormal resting ECG and/or overt heart failure had a poor prognosis in comparison individuals in control groups.^51^

In 1986, Combellas et al.^52^ studied patients with chronic Chagas heart disease without heart failure, and showed that patients with this condition were found to have diastolic abnormalities in the M-mode echocardiography. They suggested that such diastolic abnormalities might precede systolic compromise in patients with Chagas heart disease.^52^ In 1988, Marin-Neto et al.^53^ showed right ventricular systolic dysfunction in Chagas asymptomatic patients with no other evidence of left ventricular compromise, thus suggesting that the process of myocardial damage starts in the right ventricle in this disease.^53^

## The nineties

With the introduction of statistical methods into clinical practice to determine independent predictors of mortality, outcomes of patients with chronic Chagas heart disease could be better evaluated. In 1991, Espinosa and associates applied the Cox proportional hazard models to predict survival in 66 patients who were evaluated invasively and noninvasively. They demonstrated that systolic blood pressure, atrial fibrillation, cardiomegaly in the chest X-Ray, and the left ventricular end-diastolic pressure determined by left heart catheterization were independent predictors of mortality for patients with this condition.^54^ Nevertheless, when left ventricular ejection fraction was determined noninvasively by echocardiography, it was found to be the powerful independent predictor of all-cause mortality for patients with chronic Chagas heart disease.^55^

In those patients with chronic heart failure secondary to chronic Chagas heart disease, left ventricular ejection fraction and VO2 max remained independent predictors of all-cause mortality.^56^ Ventricular tachycardia induced by exercise stress testing was found to be a predictor of sudden cardiac death in patients with chronic Chagas heart disease, confirming in laboratory the association of malignant ventricular arrhythmia and sudden cardiac death. Independent predictors of sudden cardiac death not associated with physical exercise were established in 1996.^57-58^

Another important discovery from the nineties was that of the neurohormonal system activation in patients with chronic heart failure secondary to chronic Chagas heart disease. Thus, it was clear that both the Renin-Angiotensin-Aldosterone System^59^ as well as the Autonomic Nervous System^60,61^ were overactivated, similarly to what occurred in patients with non-Chagas disease heart failure, thus opening the rationale for the treatment of patients with chronic Chagas heart disease with chronic heart failure.

In 1995, Kalil et al.^62^ showed a good correlation between Resonance Magnetic Imaging and endomyocardial biopsy to detect underlying myocarditis in patients with chronic Chagas heart disease, thus heralding the potential role of such method in the study of patients with this condition.^62^ Another important finding obtained in this decade was that prognosis of outpatients with chronic Chagas heart disease was poorer than that of other types of dilated cardiomyopathy.^63^ In 1999, Rabinovitch et al.^64^ observed that the rate of recrudescence of malignant ventricular arrhythmia was 85% in patients with chronic Chagas heart disease, survivors of cardiac arrest, treated with implantable cardioverter-defibrillator. Therefore, they demonstrated the mechanism of sudden cardiac death in patients with this condition, in contrast to the role of AV block as a cause of sudden cardiac death at Chagas' time.^64^

## The 2000's

Barros et al.^65^ showed abnormalities in the isovolumic contraction time in patients with normal ECG, normal chest X-Ray, and normal echocardiogram.^65^ The same authors also demonstrated right ventricular compromise in patients with otherwise normal echocardiogram.^66^ Rochitte et al.^67^ first quantified myocardial fibrosis by Magnetic Resonance Imaging in patients with chronic Chagas heart disease. They observed that myocardial fibrosis was present in 85% of patients with this condition, and that left ventricular ejection fraction was inversely correlated to myocardial fibrosis, thus detecting in vivo what Chagas had detected in postmortem examination. Importantly, they also observed that myocardial fibrosis was present in all patients with ventricular tachycardia, which might suggest a role for the fibrosis in the pathogenesis of malignant arrhythmias in patients with this condition, as observed in non-Chagas patients.^67^

**Figure 2:**
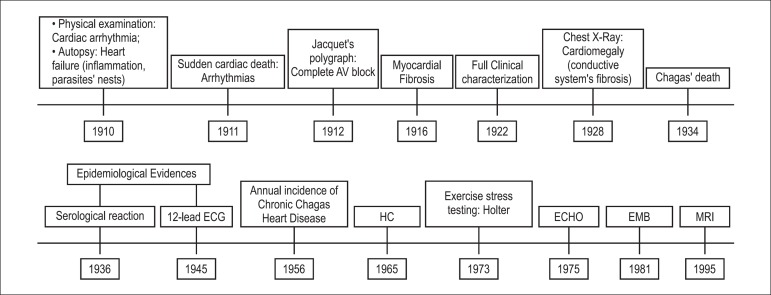
Timeline of scientific construction of the Chronic Chagas Heart Disease. HC: Heart Catheterization; ECHO: Echocardiography; EMB: Endomyocardial Biopsy; MRI: Magnetic Resonance Imaging.

The timeline of the scientific construction of Chronic Chagas Heart Disease is depicted in Figure 2.

## Conclusion

This historical reappraisal shows that Carlos Chagas' discoveries served as a backdrop to the scientific construction of chronic Chagas heart disease, and the scientific evolution that occurred with time added to his work, but, in essence, confirmed his assumptions on this formerly continental tragedy, but now a globalized disease. It is unbelievable that such a discovery did not win a Nobel Prize.^68,69^
